# A Rare Presentation of Brucellosis as Myocarditis

**DOI:** 10.7759/cureus.11345

**Published:** 2020-11-05

**Authors:** Shaheen Bhatty, Pawan Kumar, Bareera Javed, Amara Zafar

**Affiliations:** 1 Internal Medicine, Dr. Ruth K. M. Pfau Civil Hospital Karachi, Karachi, PAK

**Keywords:** brucellosis, myocarditis

## Abstract

Brucellosis is commonly transmitted by consumption of unpasteurized dairy products and most commonly presents as fever, arthralgia, fatigue, hepatosplenomegaly, and peripheral arthritis. Our patient presented with a history of three months of high-grade fever, undocumented significant weight loss, jaundice, and hepatosplenomegaly. On the seventh day of admission, he developed sudden onset of shortness of breath, and his jugular venous pressure was raised with fine crackles at the lung bases bilaterally and pedal edema up to the ankles bilaterally. Electrocardiography was done, which showed T wave inversions in 1 and augmented Vector Left (aVL). Troponin I was raised at that time, and echocardiography revealed an ejection fraction of 40%. A diagnosis of myocarditis secondary to brucellosis was made.

## Introduction

Brucellosis is a disease with a predilection toward multiple systems. It encompasses nonspecific symptoms that usually occur within two weeks to three months after the bacteria is inoculated [1]. It is still endemic in Pakistan [2]. It is commonly transmitted by consumption of unpasteurized dairy products and most commonly presents as fever, arthralgia, fatigue, hepatosplenomegaly, and peripheral arthritis [3]. Brucellosis may involve any organ or tissue in the body. However, it rarely involves cardiovascular system occurring in only 0%-2% of patients, and when involved, endocarditis is the most common manifestation. Other cardiac complications include pericarditis and myocarditis [4]. However, hepatosplenomegaly is the hallmark sign on clinical examination [5].

## Case presentation

A 30-year-old married male, with no known co-morbidities, presented to the emergency department of a tertiary care hospital with complaints of fever for three months and a history of significant undocumented weight loss. He had used multiple antibiotics from different clinics and antimalarial agents for fever in this three-month period.

On examination, his blood pressure was 120/80 mm Hg, pulse was 100 beats per minute, temperature was 101-degree Fahrenheit, and respiratory rate was 26 per minute. On general physical examination, he was jaundiced, and there was hepatosplenomegaly. On cardiac examination, there were no murmurs, rubs, or gallops, and on examination of respiratory system, lungs were clear on percussion and auscultation.

Laboratory tests revealed a low platelet count of 99,000 k/μL and raised aminotransferase of 165 μ/L; erythrocyte sedimentation rate (ESR) was significantly raised at 65 mm/hour, and C-reactive protein (CRP) was 101 mg/L. Other investigations showed a hemoglobin (Hb) of 10.9 g/dL, hematocrit 31.8%, mean corpuscular volume (MCV) 79.9%, white blood cells (WBC) 4.7 g/dL, neutrophil 64.6%, lymphocytes 24.8%, total bilirubin 6.36 mg/dL, direct bilirubin 5.98 mg/dL, alanine aminotransferase (ALT) 165 U/L, and alkaline phosphatase (ALP) 350 U/L. Peripheral film showed anisocytosis and poikilocytosis with reticulocyte count of 0.6%.

His viral markers, human immunodeficiency virus (HIV) serology, and gastric lavage for acid fast bacilli (AFB) came out negative. Two blood cultures taken 12 hours apart were negative. Urine culture and sensitivity showed no bacterial growth. Chest x-ray and echocardiography were normal.

**Figure 1:**
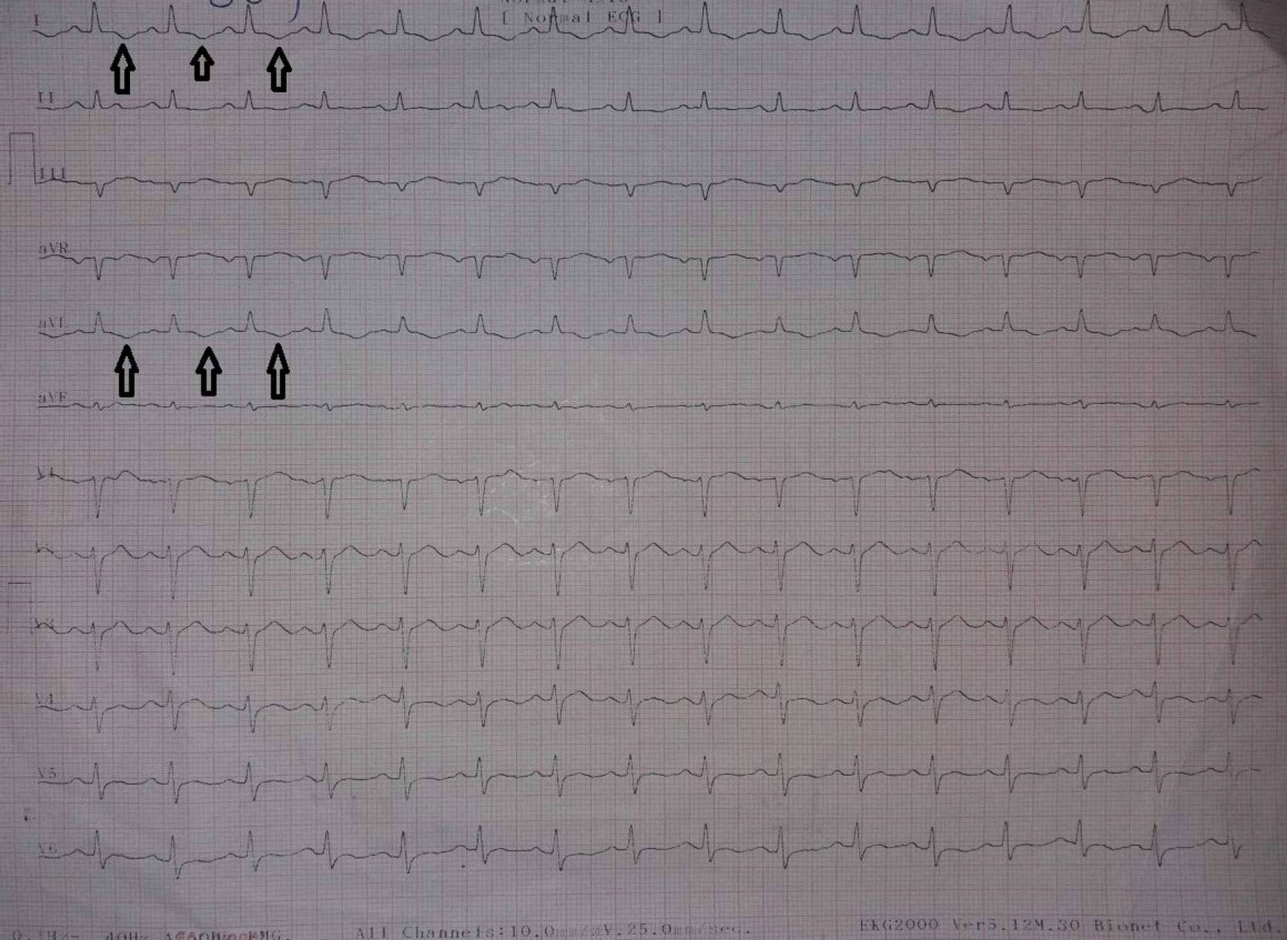
Electrocardiogram of the patient on the seventh day of his admission

During his hospital stay, on seventh day of his admission, the patient developed an episode of sudden onset of shortness of breath. On examination, jugular venous pressure (JVP) was raised with fine crackles at bilateral lung bases, and he started developing bilateral pedal edema up to the ankles. Electrocardiogram (ECG) was done that showed T wave inversions in leads 1 and augmented Vector Left (aVL) as can be seen in Figure 1. Troponin I was 3.5 ng/ml. At that time, echocardiography was done that showed ejection fraction (EF) of 40%. A diagnosis of myocarditis was made.

At that time, brucella serology was sent as the patient continued to have fever spikes, and he also revealed a history of drinking unpasteurized milk. Result was significant with titers of >1:320 for *Brucella abortus* (normal range < 1:80) and >1:160 for *Brucella melitensis* (normal range < 1:80). ESR and CRP were repeated at this time and were 80 mm/hour and 105 mg/dl, respectively. A final diagnosis of brucellosis with myocarditis was made.

The patient was started on treatment with injection streptomycin 1-gram intramuscular once daily for two weeks, 100-mg doxycycline tablet twice daily for six weeks, and two tablets of rifampicin 900 mg in the morning for six weeks. Patient’s fever subsided, and laboratory investigations were repeated after two weeks of treatment and were as follows: Hb 12.3 g/dL, MCV 84.8%, WBC 5.5 g/dL, neutrophils 51.8%, lymphocytes 34.8%, platelet count 344,000 k/uL, total bilirubin 2.81 mg/dL, ALT 45 U/L, and ALP 326 U/L.

## Discussion

Brucellosis rarely involves the heart. If cardiovascular system is involved in brucellosis, endocarditis is the most common manifestation. Myocarditis is a rare complication of adult brucellosis and in most instances is ECG and/or echocardiographic diagnosis [6]. Very few cases of brucella-related myocarditis are reported in literature. Our case highlights and adds to the deficient literature of the occurrence of myocarditis secondary to brucella infection.

In our case the patient presented with a three-month history of fever and at the time of presentation was jaundiced with hepatosplenomegaly. In 2011 a similar case was reported where a diagnosis of brucella-related myocarditis was made in a 17-year-old male after a six-day history of high-grade fever and chest pain. He also had hepatosplenomegaly at presentation like our patient [7]. ECG of our patient revealed T wave inversions in lead 1 and aVL with a positive troponin test, and ECG of the patient presented in 2011 case showed T wave inversions in lead II, III, aVF, and V2 to V6 leads. His troponin test was also positive [7].

In literature we see another case reported in 2007 where a 26-year-old man, shepherd by profession, presented with a three-month history of fever, ataxia, and dysarthria. He was diagnosed with brucellosis by positive blood cultures and had developed myocarditis with low agglutinating titers secondary to brucella infection. Regarding myocarditis his ECG showed sinus bradycardia and high T waves in the precordial leads, and echocardiography showed septal hypokinesia and EF of 30% [8]. In another case, brucella-related myocarditis was diagnosed in a 32-year-old man who presented with the complaints of acute chest pain and a night fever. His ECG was normal at presentation, but CRP and troponin I were raised. His EF was 65%. The suspicion of myocarditis here was confirmed with a cardiac magnetic resonance imaging (MRI) [9]. Myocarditis secondary to brucella infection was also reported very recently in a 21-year-old Caucasian man who was admitted with complaints of fever, fatigue, and retrosternal pain. His ECG showed ST elevation in I, II, aVL, and V4-V6, and echocardiography showed wall motion abnormalities [10].

Most studies have based the diagnosis of brucella-related myocarditis on clinical suspicion, demographic and epidemiological characteristics, symptoms of disease, and isolation of microorganisms by blood culture and serology. While pericardiocentesis and heart biopsy are required to make a definite diagnosis of cardiac involvement, these are interventional procedures that are expensive and not easily performed in routine practice and can be avoided when a safe diagnosis can be made based on other means. Our patient was indigent and declined additional studies including cardiac MRI or a heart biopsy.

Patients suffering from brucella-related myocarditis usually respond well to the antibiotic therapy. Our patient was treated with a triple antibiotic regimen; doxycycline, rifampicin, and streptomycin, and his symptoms regressed rapidly after initiation of treatment. This triple antibiotic regimen has shown similar efficacy in treating other cases of brucella-related myocarditis in literature [7,9]. In literature we see a case of brucellosis, which was complicated by severe myocarditis and resulted in death [11]. In countries like Pakistan where brucellosis is still endemic, it should be considered by the health-care professionals in all the cases presenting with prolonged fever. Timely diagnosis of brucella-related myocarditis and administration of appropriate antibiotic therapy can be life-saving for the patient. 

## Conclusions

Brucellosis is still endemic in Pakistan. It should be considered in the assessment and evaluation of febrile patients especially when presenting with nonspecific symptoms. Cardiovascular complications of brucellosis although rare should be considered as a potentially causative agent of myocarditis especially in endemic countries. Echocardiographic study should be considered if a patient tests positive for brucella. There is clinical suspicion of cardiac involvement as patients with brucella myocarditis usually respond to antibiotic therapy well, and this could prove to be life-saving for the patient. 
